# Low Grade Gliomas in Eloquent Locations – Implications for Surgical Strategy, Survival and Long Term Quality of Life

**DOI:** 10.1371/journal.pone.0051450

**Published:** 2012-12-10

**Authors:** Asgeir S. Jakola, Geirmund Unsgård, Kristin S. Myrmel, Roar Kloster, Sverre H. Torp, Sigurd Lindal, Ole Solheim

**Affiliations:** 1 Department of Neurosurgery, St.Olavs University Hospital, Trondheim, Norway; 2 MI Lab, Norwegian University of Science and Technology, Trondheim, Norway; 3 Department of Neuroscience, Norwegian University of Science and Technology, Trondheim, Norway; 4 National Centre for 3D Ultrasound in Surgery, Trondheim, Norway; 5 Department of Pathology, University Hospital of Northern Norway, Tromsø, Norway; 6 Department of Ophthalmology and Neurosurgery, University Hospital of Northern Norway, Tromsø, Norway; 7 Department of Laboratory Medicine, Children’s and Women’s Health, Norwegian University of Science and Technology, Trondheim, Norway; Cedars-Sinai Medical Center, United States of America

## Abstract

**Background:**

Surgical management of suspected LGG remains controversial. A key factor when deciding a surgical strategy is often the tumors’ perceived relationship to eloquent brain regions

**Objective:**

To study the association between tumor location, survival and long-term health related quality of life (HRQL) in patients with supratentorial low-grade gliomas (LGG).

**Methods:**

Adults (≥18 years) operated due to newly diagnosed LGG from 1998 through 2009 included from two Norwegian university hospitals. After review of initial histopathology, 153 adults with supratentorial WHO grade II LGG were included in the study. Tumors’ anatomical location and the relationship to eloquent regions were graded. Survival analysis was adjusted for known prognostic factors and the initial surgical procedure (biopsy or resection). In long-term survivors, HRQL was assessed with disease specific questionnaires (EORTC QLQ-C30 and BN20) as well as a generic questionnaire (EuroQol 5D).

**Results:**

There was a significant association between eloquence and survival (log-rank, p<0.001). The estimated 5-year survival was 77% in non-eloquent tumors, 71% in intermediate located tumors and 54% in eloquent tumors. In the adjusted analysis the hazard ratio of increasing eloquence was 1.5 (95% CI 1.1–2.0, p = 0.022). There were no differences in HRQL between patients with eloquent and non-eloquent tumors. The most frequent self-reported symptoms were related to fatigue, cognition, and future uncertainty.

**Conclusion:**

Eloquently located LGGs are associated with impaired survival compared to non-eloquently located LGG, but in long-term survivors HRQL is similar. Although causal inference from observational data should be done with caution, the findings illuminate the delicate balance in surgical decision making in LGGs, and add support to the probable survival benefits of aggressive surgical strategies, perhaps also in eloquent locations.

## Introduction

The clinical course for patients with diffuse infiltrating low-grade gliomas (LGG) is often unpredictable. In such WHO grade II gliomas, negative prognostic factors for survival are advanced age, large tumor size, midline involvement, the presence of neurologic deficits and astrocytoma histopathology [1]. In long term survivors, health related quality of life (HRQL) is often reduced, even in patients with stable disease [2].

Surgical management of suspected LGG is controversial [3]–[7]. A key factor when deciding on the surgical strategy is often the tumors’ perceived relationship to so-called eloquent brain regions, meaning involvement of sensorimotor regions, language cortices, basal ganglia and/or larger white matter tracts. LGGs located within or in close proximity to these areas may be subject to a less aggressive surgical strategy to avoid acquired motor or verbal deficits, while hopefully preserving patients’ HRQL [7]. As radical resections are less often achieved in such regions, eloquence is perhaps also a negative prognostic factor with respect to survival [8]. Despite the key importance for clinical decision making, the association between lesion eloquence for survival and long-term HRQL has not been much explored in LGGs.

In a consecutive population based series of patients with supratentorial LGG treated at two centers we aimed to study if eloquence in tumor location has implications for survival or quality of life. Our secondary aim was to provide long-term data on HRQL in LGG patients.

## Methods

### Ethics Statement

The study protocol was approved by the Regional Ethical Committee for Health Region Mid-Norway. The need for informed consent from participants in the retrospective part of the study was waived by the Committee. All patients included in the quality of life analyses have given their written and informed consent.

### Patient Selection

Patients were included from two university hospitals in Norway (University Hospital of Northern Norway and St.Olavs University Hospital). Adult patients, 18 years or older with histological verified supratentorial LGG diagnosed in the 12-year period from 1998 through 2009 were screened for inclusion. These patients were retrospectively identified from the pathology databases of the respective hospitals. Only patients with supratentorial grade II glioma (diffuse astrocytomas, oligodendrogliomas and oligoastrocytomas) were included in the study. The tumors were classified according to the WHO classification system. [9] A neuropathologist from each hospital reviewed all LGG diagnosed at the other hospital. In total, 169 cases were reviewed. The neuropathologists were blinded for previous diagnosis, baseline characteristics, image data and clinical outcomes. Initial diagnoses and review diagnoses were discordant with respect to concluding with grade II glioma in 46 (27%) of cases. The neuropathologists were given case-numbers with discordant results for a final consensus meeting where consensus was obtained by evaluation of the slides in a multi-headed microscope.

### Clinical Characteristics and Treatment

Patient and treatment characteristics were based on retrospective review of medical records. We used the system suggested by Sawaya *et al* when grading lesions with respect to anatomical eloquence [10]. In the 11 patients (7%) where preoperative images were unavailable for review we used subsequent images or the radiology reports for grading eloquence. Still, in 4 patients (2%) a highly reliable grading was not achieved and these patients were excluded from the analyses involving eloquence. In the cases where preoperative images couldn’t be evaluated we imputed the tumor diameter using the mean tumor diameter from the treating department. To adjust for important prognostic factors in LGG we used the Pignatti score (age ≥40 years, diameter ≥6 cm, crossing midline, deficit present and astrocytoma histology) [1]. Medical important co-morbidity was scored with Charlson Co-morbidity Index (CCI) [11]. No patients were excluded based on radiographic findings or clinical status.

Surgical resections were carried out with use of the Stealth® neuronaviagion system (Medtronic, Minnesota, US) with or without 2D ultrasonography at University Hospital of Northern Norway. At St.Olavs University Hospital the SonoWand® neuronavigation system (SonoWand, Trondheim, NO) with integrated 3D-ultrasonsography was used with incorporation of functional data (functional MRI and/or diffusion tensor imaging) in selected eloquent lesions. Cortical mapping or awake surgery was not utilized in any patients. Adjuvant therapy was usually administered with surgically untreated tumors or with progressive disease or signs of malignant transformation.

### Follow-up

All patients were followed until death or until April 11^th^ 2011. The national population registry (Statistics Norway) provided the patients’ current status (dead/alive) as of 11^th^ April 2011 and the date of death. No patients were lost to follow-up for survival analysis. Patients who were still alive were contacted by mail with a request to participate in the HRQL part of the study and to answer HRQL questionnaires. Non-responders received one remainder by mail.

### Health-related Quality of Life

#### EORTC QLQ-C30

QLQ-C30 consist of cancer specific functional and symptom scales in addition to a scale on overall health and HRQL [12]. The questionnaire also contains single-items, but none were explored in this study. Possible answers range from 1–4, where 1 is described as “not at all”, 2 as “a little”, 3 as “quite a bit”, and 4 as “very much”. This applies for all but two questions on global health status where the scale range from 1–7. All domains are convertible to a score (0–100). A high score represents a high HRQL and level of functioning and in the symptom scales a higher score indicates more symptoms. We assessed the overall HRQL, cognitive function and fatigue only. The other items and domains in the questionnaire were not explored.

#### EORTC QLQ-BN20

This questionnaire is specifically designed for brain cancer patients [13]. The QLQ-BN20 consists of four multi-item scales (domains): future uncertainty, visual disorder, motor dysfunction, and communication deficit. Scores in the domains are converted to a scale (0–100) where higher score represents *worse* HRQL. We assessed all four domains. The questionnaire also consists of seven single items in which none were explored in this study.

#### The EuroQol 5D

EQ-5D is a generic and preference-weighted measure of health-related quality of life [14]. The questionnaire has been applied to a wide range of health conditions and treatments. The EQ-5D has also been validated in a Norwegian population [15]. The EQ-5D questionnaire is a generic HRQL instrument. In EQ-5D five dimensions of HRQL are scored: mobility, self-care, usual activities, pain/discomfort and anxiety/depression with 3 possible answers to each dimension. This results in the 243 different possible health states which are transformed into an index value based on a large survey in the UK population [16]. EQ-5D index value is from –0.594 to 1, where 1 corresponds to perfect health, and 0 to death. Negative values are considered to be worse than death. A visual analogue scale where patients rate their current health state on a line ranging from 0–100 (worst to best imaginable health) forms the second part of the EuroQol questionnaire. In this study only the index value was assessed.

### Statistics

All analyses were done with PASW, version 18.0. Central tendencies are presented as means ± SD. Independent samples t-test was used for comparisons of means between groups. Survival analysis assessing eloquent location was analyzed with log-rank test and presented with Kaplan-Meier curves. The estimated 5-year survival rates were created using life tables. In addition we created a Cox multivariable model adjusting for the Pignatti score. For the specific questions in the EORTC questionnaires we dichotomized the items (‘not at all’ and ‘a little’ against ‘quite a bit’ and ‘very much’) within the domains for descriptive analyses. Among possible HRQL subscales we explored only the domains communication, motor function, visual function, future uncertainty and cognitive function as well as more global categories (fatigue and global HRQL). Statistical significance level was set to p≤0**.**05. All tests are two-sided.

## Results

**Figure 1:**
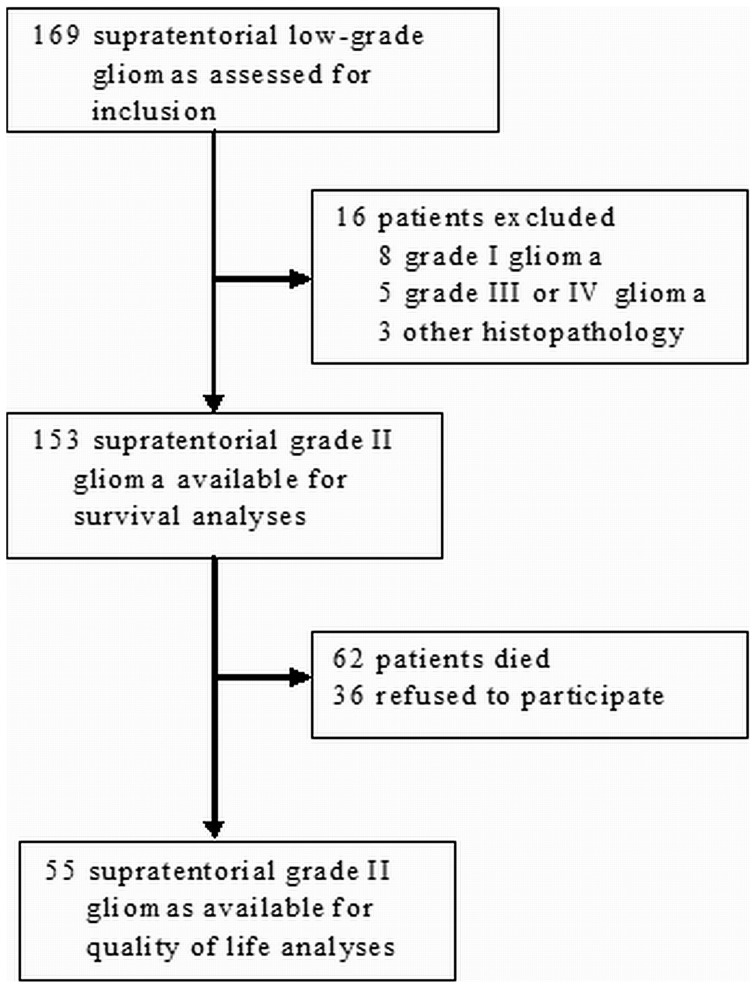
Flow-chart describing patient selection.

After the central review of initial histopathology in all LGG, 153 adults with supratentorial LGG were identified and included in the study (figure 1). In 46 patients (31%) the tumor was located in an eloquent area. Among the 46 patients with LGG in eloquent locations, 22 (48%) underwent biopsy only as the initial surgical procedure, as compared to 17 (29%) in non-eloquent lesions. Baseline characteristics for the different groups are shown in table 1.

**Table 1:** Baseline characteristics.

Baseline	Non-eloquent (N = 59)	Intermediate (N = 44)	Eloquent(N = 46)	With HRQL_†_ (N = 55)
Age ± SD	42±15	43±14	48±17	41±13
Age ≥40 years	29 (49%)	23 (52%)	29 (63%)	26 (47%)
Female	29 (49%)	19 (43%)	15 (33%)	25 (45%)
Preoperative KPS ≥80	54 (92%)	35 (80%)	31 (67%)	50 (91%)
Charlson co-morbidity index, mean ± SD	0.12±0.38	0.25±0.53	0.22±0.66	0.13±0.34
Treatment year, mean ± SD	2003±3	2004±3	2004±4	2004±3
Maximal tumor diameter, mm ± SD	39±14	50±15	64±19	43±16
Tumor diameter ≥60 mm	4 (7%)	12 (27%)	27 (59%)	8 (15%)
Midline or bilateral involvement	1 (2%)	8 (18%)	12 (26%)	1 (2%)
Preoperative contrast enhancement	7 (12%)	9 (21%)	11 (24%)	6 (11%)
Pignatti score				
0	6 (10%)	2 (5%)	–	7 (13%)
1	26 (44%)	11 (25%)	13 (28%)	25 (45%)
2	23 (39%)	16 (36%)	9 (20%)	17 (31%)
3	3 (5%)	11 (25%)	13 (28%)	5 (9%)
4	1 (2%)	4 (9%)	6 (13%)	1 (2%)
5	–	–	5 (11%)	–
Initial surgical procedure				
Biopsy	17 (29%)	19 (43%)	22 (48%)	10 (18%)
Resection	42 (71%)	25 (57%)	24 (52%)	45 (82%)
Histopathology				
Astrocytoma	44 (75%)	35 (80%)	35 (76%)	29 (53%)
Oligodendroglioma	9 (15%)	6 (14%)	6 (13%)	16 (29%)
Oligoastrocytoma	6 (10%)	3 (7%)	5 (11%)	10 (18%)
Initial symptoms_§_				
Seizure	40 (68%)	33 (75%)	30 (65%)	33 (60%)
Seizure only	38 (64%)	26 (59%)	26 (57%)	31 (56%)
Headache	13 (22%)	13 (30%)	12 (26%)	12 (22%)
Motor	2 (4%)	4 (9%)	7 (15%)	1 (2%)
Cognitive	4 (7%)	7 (16%)	11 (24%)	8 (15%)
Dysphasia	1 (2%)	–	2 (4%)	1 (2%)
Asymptomatic	2 (4%)	1 (2%)	1 (2%)	2 (4%)

* KPS = Karnofsky Performance Status ^¶^Eloquent graded as defined by Sawaya. [10] ^§^Several symptoms may be registered per patient. ^†^HRQL = Health-related quality of life. There were four missing values with respect to grading of eloquence.

### Survival

**Figure 2:**
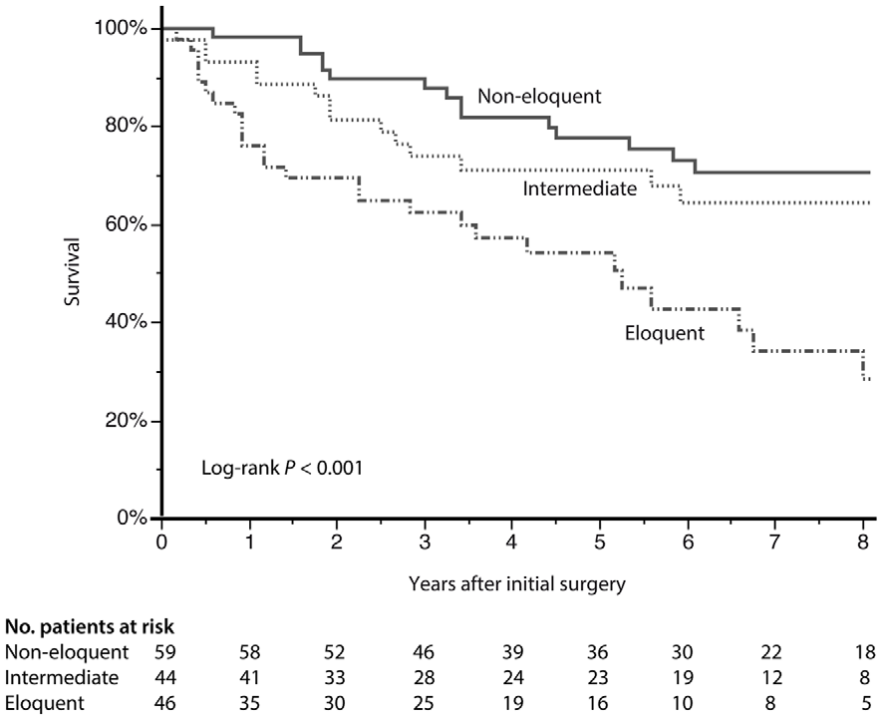
Association between eloquence and survival (N = 149). A decreased survival was seen with closer distance of tumor to sensitive regions as demonstrated with the Kaplan-Meier curve (p<0.001, log-rank test). The estimated 5-year survival was 77% (95% CI 65–89) in non-eloquent tumors, 71% (95% CI 57–85) in intermediate located tumors and 54% (95% CI 38–70) in eloquent tumors.

Treatment and disease characteristics are demonstrated in table 2. There was 8 patients (13%) among the 62 diseased who did not receive any adjuvant therapy during the course of the disease (terminal condition 4, early death 2 (22 days and 33 days), refused therapy 1, unrelated cause of death with stable disease 1). The main reason for not receiving adjuvant therapy among the 91 patients still being alive at end of follow-up was stable disease in 33 patients (36%). As shown in figure 2, there was a significant association between eloquence and survival (log-rank, p<0.001). The estimated 5-year survival was 77% (95% CI 65–89) in non-eloquent tumors, 71% (95% CI 57–85) in intermediate located tumors and 54% (95% CI 38–70) in eloquent tumors. Median survival was 116 months for the entire group (95% CI could not be calculated from the material). Median survival in patients with eloquent lesions was 5.3 years (95% CI 3.0–7.5) while it was not reached for the other groups. In the Cox multivariable model we included the Pignatti score to adjust for important (and skewed, see table 1) baseline parameters in addition to location (non-eloquent, intermediate, eloquent). In this adjusted analysis a significant survival difference was still seen with a relative hazard ratio of 1.5 (95% CI 1.1–2.0, p = 0.022) with closer distance to eloquent areas. The Pignatti score proved its predictive capabilities in the same analysis (p<0.001, hazard ratio 1.7, 95% CI 1.3–2.1).

**Table 2:** Treatment and disease characteristics after initial surgery.

	Non-eloquent (N = 59)	Intermediate (N = 44)	Eloquent (N = 46)	With HRQL_†_ (N = 55)
Surgical complications	2 (4%)	4 (9%)	6 (13%)	4 (7%)
New or worsened neurological deficits*	9 (15%)	12 (27%)	8 (17%)	14 (26%)
Early (first 6 months) postoperative radiotherapy	16 (27%)	16 (36%)	21 (46%)	14 (26%)
Ever radiotherapy	30 (51%)	33 (75%)	35 (76%)	25 (46%)
Early (first 6 months) postoperative chemotherapy	7 (12%)	12 (27%)	12 (26%)	9 (16%)
Ever chemotherapy	21 (36%)	28 (64%)	28 (61%)	18 (33%)
Later/repeated resection	15 (25%)	18 (41%)	15 (33%)	12 (22%)
Malignant transformation_§_	18 (31%)	24 (55%)	25 (54%)	10 (18%)

* Documented neurological deterioration of *any* magnitude in the postoperative course. ^§^Malignant transformation if verified new contrast enhancement or malignant histology from new biopsy or resection. ^†^HRQL = Health-related quality of life. There were four missing values with respect to grading of eloquence.

### Long-term Quality of Life

As of April 11^th^ 2011 91 patients (59%) were still alive. There were 36 (40%) non-responders. The non-responders had higher Pignatti scores (p = 0.001) and Charlson Co-morbidity Index (p = 0.007), but presented with seizures preoperatively as often as responders (p = 0.359). Among living patients, 55 (60%) were willing to participate in the HRQL study and returned the questionnaires. Their baseline characteristics are presented in table 1. In 30 (65%) patients the location of tumor was non-eloquent, in 12 (22%) intermediate and in 12 (22%) eloquent. The mean follow-up among questionnaire responders was 7.2 years (range 1.6–13.2). The long-term HRQL data from the 55 LGG survivors is presented in table 3.

**Table 3:** Eloquence in relation to quality of life scores.

	Non-eloquent* (N = 30)	Eloquent* (N = 24)	p-value
BN20: Communication deficit	17	21	0.481
BN20: Motor dysfunction	18	23	0.422
BN20: Future uncertainty	21	23	0.721
QLQ-C30: Fatigue	31	33	0.850
QLQ-C30: Cognitive function	68	69	0.957
QLQ-C30: Overall quality of life	72	74	0.837
EQ-5D: Index value	0.76	0.74	0.785

* Non-eloquent is here grade 1 defined by Sawaya [10], while eloquent here denotes grade 2 *and* 3 (intermediate and eloquent). This grouping was done to achieve adequate number of patients in each group. There was one missing value with respect to tumor location.

### Brain Tumor and Cancer Specific Quality of Life

**Figure 3:**
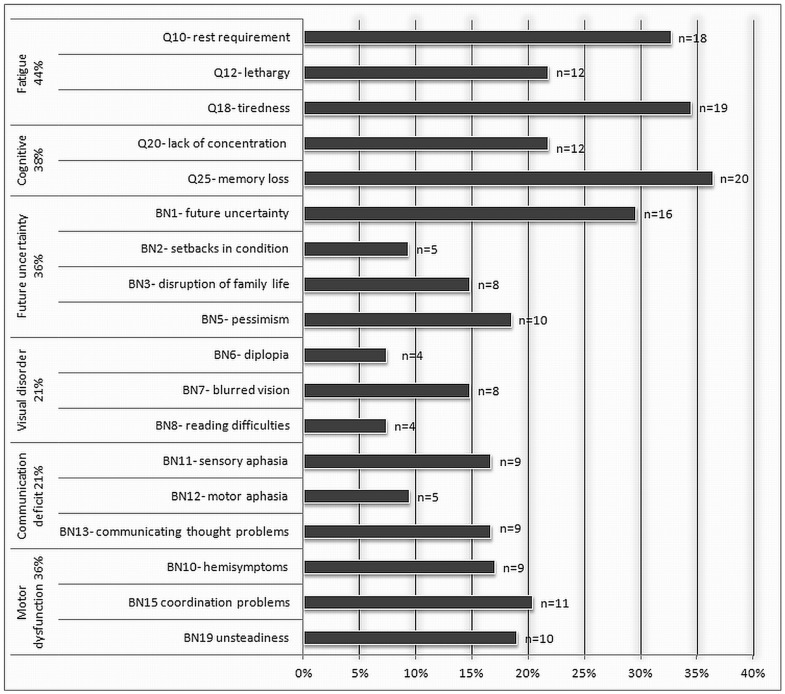
Specific symptoms within domains (N = 55). Percentage in domains represents patients with one or more symptom within category. Only patients with “quite a bit” and “very much” symptoms are presented.

A descriptive analysis of patients with high symptom burden within the HRQL domains that were explored in this study are presented in figure 3. The fatigue domain was most often affected as 44% experienced symptoms in at least one of the fatigue related questions. Other frequent complaints were memory loss (36%) followed by tiredness (35%), rest requirement (33%) and uncertainty concerning the future (29%). As only one patient had a high symptom burden with respect to seizure activity, seizures were not included it in the analyses. The internal consistency measured with Cronbach’s alpha was between 0.80–0.94 for all domains except “visual disorder” with 0.60, and as a consequence it was excluded from between group comparisons.

### Eloquence and Long-term Quality of Life

We analyzed functions and overall HRQL in patients with non-eloquent lesions compared to patients with lesions in intermediate and eloquent locations (table 3). For categories in the BN20 module the differences between groups were not significant for any of the explored items (communication deficit, motor dysfunction, and future uncertainty). There were also no statistical significant differences in cognitive function, fatigue, overall HRQL, and EQ-5D index value.

## Discussion

In the present study, we found impaired survival in patients with LGGs in eloquent locations compared to patients with tumors in other areas. The impact of eloquence on survival remained after adjusting for established prognostic factors that were skewed at baseline. In long-term survivors, reported HRQL was not statistically different in patients with eloquent and non-eloquent lesions.

In our study of 153 consecutive patients, 30% of all LGGs were defined as eloquent in location based on the Sawaya classification system [10]. However, to be remembered, eloquence as defined anatomically [10] does not always correlate to functional regions as characterized with various techniques, such as functional MRI or direct cortical stimulation. It was for example recently reported that presumed eloquence from imaging can be a modifiable risk factor for impaired survival since eloquent LGG can still be safely removed in many instances [8]. Perceived eloquence is still often of key importance when neurosurgeons decide on the surgical strategy with respect to aggressiveness and invasiveness of the procedure in addition to planning of which techniques or technology to utilize. This was demonstrated in our study by the declining percentage of resections with increasing eloquence of the tumor. However, is eloquence as important for the patients as it is for the surgeons? Neurosurgeons have tradition for a special respect for areas perceived of so-called eloquence with a traditional focus on the executive motor and language regions, even though there are presumably no non-functional regions in the brain. Much less attention and respect is given to brain regions that may affect various cognitive functions, personality functions or sensory neglect. Is the discrimination against other brain functions justified? In unselected patients with gliomas we earlier observed that new neurologic deficits have an immense effect on short-term HRQL [17]. Although a paresis may perhaps seem more important than the mild cognitive deficit in the short term, this may be different in the long term. How different surgically induced deficits affect HRQL over time is largely unknown, and how the response shift that may occur over time affects results remains speculative [18]. In a recent cross-sectional study of long term HRQL in LGGs, Aaronson *et al* found that most of the impairment in HRQL was attributed to the cancer diagnose per se, but with an additional burden in cases with neurocognitive symptoms and poor seizure control. While motor deficits were rare, approximately one quarter of patients reported serious problems with neurocognitive functioning, particularly memory and concentration [2]. This is supported by the findings in our study with a relatively high symptom burden in several assessed HRQL domains, especially for cancer related symptoms and less pronounced in specific brain symptoms including functions associated with eloquent location. Except from seizures, that are known to lower HRQL, [2] the disease specific symptom burden was largely similar to previous studies using the EORTC QLQ-BN20 questionnaire (table 4). However, concerning future uncertainty somewhat lower scores were noted in the LGG studies at long-term follow-up compared to high-grade gliomas recently diagnosed. If this reflects the difference in aggressiveness of the disease or the time since diagnose or both remain unknown. Seizures and use of antiepileptic drugs are well-known risk-factors for impaired HRQL and cognition [19]–[22]. It seems like ongoing seizure activity is related to impaired HRQL, while use of antiepileptic drugs have a stronger association with cognitive function [22]. In fact, the study from Klein *et al* suggest that patients with stable disease with grade II glioma and seizures have similar HRQL to epilepsy patients without glioma [22]. However, as only one patient had high symptom burden with respect to seizures in the present study, the impact or seizures were not explored in the HRQL analyses.

**Table 4:** A selection of glioma studies utilizing the EORTC QLQ-BN20 questionnaire.

Study	Time assessed	N	Population	CD	MD	VD	FU
Present study	Long-term (mean 7 yrs)	56	Supratentorial diffusely infiltrative grade II glioma	20	22	12	23
Klein et al [26]	Before radiotherapy	68	Consecutive newly diagnosed high-grade glioma patients eligible for radiotherapy	25	25	19	49
Taphoorn et al [13]	Before adjuvant therapy	742–745*	Two EORTC randomized trials in newly diagnosedhigh-grade glioma patients	18	18	13	37
Aaronson et al [2]	Long term (mean 5.6 yrs)	195	Dutch nationwide study in grade II gliomas,including only stable disease	24	13	15	24

The multi-item domains communication deficit (CD), motor dysfunction (MD), visual disorder (VD), and future uncertainty (FU) are reported.

Several important aspects when caring for patients with LGG were illuminated in our study. First, eloquence affects the chosen surgical strategy and seems to have an independent and dose-response effect on survival. A direct biological effect of tumor location on survival cannot be out-ruled, but the finding may also be attributed to the prognostic importance of extensive resections [23]. Thus, more widespread use of technology to aid safe and extensive resections in eloquent lesions is perhaps beneficial. Second, our findings may indicate that our surgeons have been careful enough to avoid overly aggressive treatment since neurological deterioration was infrequent in eloquent locations and since HRQL was much alike in the two groups. More aggressive resections in eloquent locations could perhaps have been favorable with respect to survival, but how long-term HRQL could have been affected by more radical surgery remains speculative. This demonstrates the typical dilemma patients and their treating neurosurgeons regularly have to deal with in glioma surgery. Third, functions in eloquent brain regions may perhaps be of less importance for patients HRQL than perceived by surgeons. Unfortunately, a study with high-quality data on longitudinal HRQL in consecutive LGG patients is still lacking. In sum, our study may suggest that a surgical strategy advocating extensive resections may outweigh the risks in most LGG. With the use of modern technology such as 3D ultrasound, intraoperative MRI [24], or mapping techniques [25] quite safe resections are most often possible, even in eloquent locations. Further refinement of tools and techniques should be encouraged to safely enhance tumor removal.

The major strengths of this study is the high external validity due to the unselected population based inclusion, the review of histopathology for the survival analysis and the adjusted survival analysis with respect to known prognostic factors. Without this adjustment the worse prognosis seen in eloquent tumors could simply have been explained by patients having worse functional status and larger tumors more often crossing the midline. In addition, the long-term HRQL data is rare in the LGG literature and offer a more holistic perspective on the tolls of the disease and its management than the traditional surgical outcome measures such as extent of resection or complication rates. However, this study has limitations as well. Unfortunately we have no information on resection grades since early MRI was not routinely performed at both hospitals. Collection of data except for HRQL was done retrospectively. However, survival is a robust end-point unaffected by the collection of data. Further, the responding patients who provided HRQL data were pre-selected by their survivorship – and those responding seemed to in the best prognostic group at time of diagnose. Thus, the long-term findings in this study may best apply to patients with good prognostic factors at presentation. Perhaps also influencing the results was the rather low statistical power in the HRQL analyses.

### Conclusion

In conclusion, patients diagnosed with supratentorial WHO grade II glioma in eloquent brain regions are less likely to undergo resections and have impaired survival compared to patients with tumors in other regions. There was no apparent association between HRQL and lesion eloquence in long-term survivors. Making causal inference from observational may be treacherous, still our findings illuminate the delicate balance in surgical decision making in LGGs, and add support to the probable survival benefits of aggressive surgical strategies, perhaps also in eloquent locations.

## Acknowledgments

We would like to thank Anne-Lise Furnes at University Hospital North Norway and Lisa M. Sagberg at St.Olavs University Hospital for facilitating this research project by helping in data collection. In addition we would like to thank Ole K. Losvik for help with figures.
